# Abstract of the Report of the Foreign Relations Committee of the National Association of Dental Faculties for the Year 1900–1901

**Published:** 1901-10-15

**Authors:** 


### ABSTRACT OF THE REPORT OF THE FOREIGN RELATIONS COMMITTEE OF THE NATIONAL ASSOCIATION OF DENTAL FACULTIES FOR THE YEAR 1900-1901.

Reported and adopted at the eighteenth annual meeting held in Milwaukee, Wis., August, 1901.

The past year has been an exceedingly active one for the Foreign Relations Committee, and the correspondence has been very large. We believe that the influence of the National Association of Dental Faculties has been materially extended during the year, and the good work that has been accomplished by it is becoming more widely known both at home and abroad.

At the last annual meeting the committee presented a partial schedule of equivalents to be given for attendance on foreign courses of study. The association accepted that and enacted that advanced standing in American schools should only be given foreign students in accordance therewith.

Your committee found that some foreign students had already been accepted by schools, and consideration given to foreign instruction which might be in conflict with the new regulations. It was therefore deemed best not to give any rulings affecting the annual term for 1900–1901.

But many schools which were most anxious to improve the standard of American professional education, have referred all their foreign applications to the committee. In this way it has been learned that foreign students have asked for advanced standing because of attendance, in some instances, on schools that had no existence whatever. Certificates have been presented from countries which have no dental legislation, and in which there is no semblance of a dental educational institution. They usually emanate from some private practitioner whose office is made to assume a sounding title. In other cases they pretend to be granted by some teaching hospital which has no official status.

Your committee has discovered that it is usual for the possessors of such doubtful credentials to write to a considerable number of schools to learn which, if any, will accept their certificates, and to find out whether some institution will not offer a special inducement. Each dean is assured that others will receive the applicant if he does not. The result is that all of those to whom application has been made are duly informed of the qualifications of suspected students and the probable terms on which they were accepted if the name is found on the lists of any school.

By this it is readily perceived how deans of colleges have been deceived in the past, and how the character of our American schools has been made to suffer for things over which they had no control. The Foreign Relations Committee is prepared to recommend a rating for any foreign school that will submit its curriculum of study and its preliminary requirements of education.

### FRAUDULENT DENTAL DEGREES.

It is not generally known in this country that thousands of fraudulent diplomas have been sold abroad. Were it possible for foreigners to distinguish between the reputable and the disreputable schools this would not so much matter, but the statutes of the State of Illinois, under which it is possible to incorporate degree-granting institutions which have practically no State supervision or responsibility whatever, and which with legal sanction, are, under the great seal of the State, certified as lawfully organized colleges by the Secretary of State, of Illinois, simply encourage the fraud. By that certification of the Secretary of State the most unblushing impostures are placed apparently upon the same plane with reputable institutions, and foreigners are deprived of all means by which they can positively determine which are worthy of recognition and which are not. As a consequence some foreign governments have used this condition, either honestly or as a desirable pretext to discriminate against all Americans, and have refused them permits to practice, and positively prohibited under heavy penalties the employment by any one of the American degree or title. This interdiction is spreading very fast, and, unless something is done to forestall it, soon the possession of an American diploma, whether legitimately or illegitimately obtained, will be a positive detriment to a practitioner. In fact, that is the case to-day in some parts of Germany. The influence of such enactments upon American educational affairs and upon the members of this association may perhaps be imagined. Already prohibition is practically accomplished in Southern Germany, is impending in Northern Germany, has been commenced in France, in Italy, and other countries, and there is sharply threatened a combination of all Europe against the American dental degree and the American dental school.

Much of this may, with a considerable degree of justice, be charged against the State of Illinois. Its own legislation has fostered the fraudulent schools that have brought this disgrace upon us. Its dental profession is not without responsibility. Not a voice has been raised in denunciation of this condition, not a word has been uttered, until a the last annual meeting of its State society a mild resolution deprecative of the infamous traffic was offered by one unconnected with either schools or boards.

The State Dental Examining Board of Illinois has practically recognized fraudulent and irregular colleges, schools either without any regular course of instruction or with but a very insufficient one, by admitting their students to the State examination and licensing them to practice, and by practically certifying to the regularity of institutions which every dentist in America should know, are conducted solely for whatever of revenue there may be in it. The law admits to the State examination for practice anyone who asks for it, and the State Board of Dental Examiners has given the known fraudulent institutions a quasi status by admitting those holding their purchased diplomas to the examination, passing them and giving them the certificate which makes of them regular and legal practitioners. This has been done under the law, but it is Illinois law, and the profession of the State is doing nothing to bring about a reform that professional decency imperatively demands. It is time that the many high-toned professional men of the State were aroused to the stern accountability to which they are liable to be called.

Last year your committee was able to report that the worst of the fraudulent schools of Chicago had been closed and that their conductors were in prison. That which was done was to a large degree the work of the State Board of Health of Illinois, which brought suit under the United States laws that forbid the use of the United States mails for fraudulent purposes. In no other way could the general government at Washington interfere. The Board of Health being a State institution, it could commence proceedings in the name of the State, and use State funds for the prosecution of the criminals. It has been appealed to by your committee to take up the fraudulent issue of dental degrees, but we have been informed by the board that it can not interfere in the dental diploma business, but will confine its work to fraudulent medical schools.

It may thus be seen that we are thrown upon our own resources in the work of closing the institutions engaged in granting fraudulent or irregular dental degrees, and can look to the medical profession for no assistance. Your committee feels confident it can within a short time close up the last of the fraudulent schools, if a sufficient sum of money can be placed at its disposal, we are so advised by very competent legal counsel. We are prepared to submit a plan of procedure to this association.

### AMERICAN EDUCATIONAL AFFAIRS IN EUROPE.

Practically no fraudulent degrees are sold in America; the countries of Europe are the sea into which the foul tide empties, and the sweetening of the waters can not be effected there. It is in this country that the remedy must be applied, and until a healthy public and professional sentiment can be evoked here nothing can be done. The condition has existed for years, and it is constantly growing worse.

### FOREIGN DENTAL SCHOOLS.

In the face of the fact that a most determined effort is being made in some foreign countries to break down the reputation of American dental schools, and to discredit all American professional education, and in the knowledge that not only are our courses refused any consideration, but sometimes made a pretext upon which to forbid Americans to enter upon practice, this association can not be accused of illiberality or of professional narrowness should it decline to accept foreign qualifications as a sufficient warrant for practice in this country. There should be some kind of reciprocity in professional affairs, and Americans ought not to be expected to extend all the professional courtesies granted. And yet, exact justice might, in the minds of many, demand that irrespective of what may be done to us, we should be forgiving, and in return for the buffetings that we receive humbly expose the other cheek to the smiting hand. That course is perhaps highly Christian, but it is not quite in accordance with the impulses of an ordinary human nature. The man or the school that does not have sufficient of self-respect to maintain inalienable rights can scarcely expect to receive the consideration which may be honestly due.

But were this the only reason to be urged against the unquestioned acceptance of all foreign qualifications, we might justly be called churlish and professionally illiberal were we to exclude any one who asked our recognition. America was the first to establish any system of dental education. It embraced a full course of instruction, the whole of which must be covered within the walls of a duly chartered institution devoted to dental instruction. It was provided that all work leading to our special degree must be done under the direct supervision of qualified and accepted teachers. Recognizing the prosthetic department as one of the most important in dental practice, we insisted that it must have a scientific basis, and not be a matter of mere empiricism. We established the principle that our students must be under the pupilage of one who was acquainted with mechanical laws, and that the teaching of physical science should not be entrusted to possible charlatans. The instructor in mechanics must be responsible to the authority which granted the diploma or certificate of qualification.

The opposite course was pursued in founding the dental system of education in some other countries. Recognizing that many skillful mechanics were outside the pale of the fully qualified men, they practically excluded prosthetics from the college curriculum, classed mere mechanical skill as handicrafture, and permitted its instruction to be received at the hands of irresponsible men. They established a system of apprenticeship which in a manner bound out the student to a dental mechanic, who should give him instruction in one of the most important departments of dentistry. It could not be expected that we should accept such instruction as the equivalent for our full college courses. This condition was the most embarrassing question that came before your committee in the attempt to establish a system of equivalents. Our schools refuse to give to an American student any advanced standing for time spent in the laboratory or office of a practitioner who has not teaching experience and responsibility. The matriculant may have passed years in a dental office, but he must join the freshman class on entering our colleges. Our diplomas or certificates are only granted upon the completion of a definite scholastic course.

Under our present legislation it is illegal and irregular for any member of this association to admit to its senior class any student who has not at least the following quallification:

Successful completion of two full terms in a dental school, *whose course has been accepted by this association as a full equivalent for its own*, and who shall by that school be recommended for such advanced standing.

Admission to the second or junior class of any of our schools can only be permitted to those who have one of the following qualifications.

1. Successful completion of one full term in a dental school whose course has been accepted by this association as a full equivalent for its own courses, the student being by that school recommended for such advanced standing.

2. Successful completion of the full course of some regular and duly accepted medical school, and graduation with the degree of Doctor of Medicine.

No partial courses are accepted, nor those spent in a school not fully and definitely recognized by this association. Surely we can not grant more than this to those making application from foreign countries while denying it to our own people.

This principle has governed the Foreign Relations Committee in making its recommendations for the recognition of foreign schools. There have been urgent requests for such recognition, but your committee has not felt itself at liberty to recommend what is not granted to our own schools and people. If any foreign school will demonstrate that its curriculum of study is the full equivalent of our own, and that it has complied with the statute of minimum requirements established by this association at its last annual meeting, your committee will be prepared to examine its claims and to recommend such action to this association as the course of study seems to warrant.

Your committee, in conclusion, points with no ordinary pride to what has been accomplished within the past five years as the result of an attempt to regulate our relations with foreign schools and foreign students, and to the high professional ground on which we now stand. There should be no further complaints, on the one hand that we accept unqualified men from abroad, or on the other that foreigners can come here and, without going through the full course demanded of American students, carry off our honors and claim to be American dentists, the colleagues of those who have completed our full curriculum of a broad course of dental study.

The foreign advisory boards, appointed with the approval of this association, have proved to be useful auxiliaries in the carrying out of our system of education. In Europe they have completed an organization, and will henceforth work together in harmony. They must exercise an important and wide influence in educational affairs, and their action can not but be for good. They will guard the interests of those holding the American degree, and help to prevent it from being unworthily conferred.

REPORT CONCERNING FOREIGN EQUIVALENTS AS AMENDED FOR THE YEAR 1901.

Were your committee to follow the precedent set by most foreign countries, no consideration would be given to their qualifications. Although America set an example to all the world in establishing a definite curriculum of instruction for dentists, in organizing schools for their theoretical and practical training, thereby erecting into a recognized profession or specialty that which previousty was mainly empiricism and charlantry, no official recognition of its special curriculum has ever been given by the dentist of foreign countries, although in great numbers they have attended our schools to obtain the advantages offered by that curriculum.

Your committee believes it to be neither fraternal, professional nor just to adopt the same course, but thinks it both expedient and right to extend proper recognition to whatever can be received as an equivalent for our own courses. It must not be forgotten, however, that the system of dental instruction in Europe varies very widely from that of our special American schools. Instruction separate from that afforded by the medical schools or universities is very rare, and the practical training which forms a part of our curriculum is usually given by private preceptors. Your committee does not feel at liberty to recommend the acceptance of an oral and theoretical course as the equivalent for one including practical work. We can not believe that the certificates of private and irresponsible practitioners can by us be accepted as any part of a college course, and hence we have given them little consideration. The report recommends that one year's credit be allowed graduates of recognized schools of England, France, Germany, Switzerland, Sweden and Austria.

